# *Lactobacillus rhamnosus* GG Lysate Increases Re-Epithelialization of Keratinocyte Scratch Assays by Promoting Migration

**DOI:** 10.1038/srep16147

**Published:** 2015-11-05

**Authors:** Walaa Mohammedsaeed, Sheena Cruickshank, Andrew J. McBain, Catherine A. O’Neill

**Affiliations:** 1 Institute of Inflammation and Repair, The University of Manchester, Manchester UK; 2 Faculty of Life Sciences, The University of Manchester, Manchester UK; 3 Manchester Pharmacy School, The University of Manchester, Manchester UK

## Abstract

A limited number of studies have investigated the potential of probiotics to promote wound healing in the digestive tract. The aim of the current investigation was to determine whether probiotic bacteria or their extracts could be beneficial in cutaneous wound healing. A keratinocyte monolayer scratch assay was used to assess re-epithelialization; which comprises keratinocyte proliferation and migration. Primary human keratinocyte monolayers were scratched then exposed to lysates of *Lactobacillus (L) rhamnosus* GG, *L. reuteri, L. plantarum* or *L. fermentum.* Re-epithelialization of treated monolayers was compared to that of untreated controls. Lysates of *L. rhamnosus* GG and *L. reuteri* significantly increased the rate of re-epithelialization, with *L. rhamnosus* GG being the most efficacious. *L. reuteri* increased keratinocyte proliferation while *L. rhamnosus* GG lysate significantly increased proliferation and migration. Microarray analysis of *L. rhamnosus* GG treated scratches showed increased expression of multiple genes including the chemokine CXCL2 and its receptor CXCR2. These are involved in normal wound healing where they stimulate keratinocyte proliferation and/or migration. Increased protein expression of both CXCL2 and CXCR2 were confirmed by ELISA and immunoblotting. These data demonstrate that *L. rhamnosus* GG lysate accelerates re-epithelialization of keratinocyte scratch assays, potentially via chemokine receptor pairs that induce keratinocyte migration.

The potential beneficial effects of the oral consumption of probiotic bacteria on intestinal health have been extensively investigated. Probiotics are usually members of the genera *Lactobacillus* or *Bifidobacterium* and may exert a positive benefit on the gut using through a variety of mechanisms, including inhibition of the growth of pathogenic bacteria^1^, epithelial repair, enhancement of the gut barrier^2^ and modulation of the immune response^3^. Due to their potential for maintaining gut health and combating disease, various species of lactobacilli have been tested in tissues other than the gut, for example the vagina and the oral cavity^4^,^5^ and there is a growing body of evidence that probiotic bacteria may also be of benefit in these tissues. Our interest has been in the development of topically applied lactobacilli as treatments for skin in health and/or disease^6^,^7^,^8^. Our focus on lactobacilli is based on the fact that in contrast to skin microbiota, lactobacilli are relatively well characterised in terms of their safety and mechanisms of action. Thus, we believe that the development of lactobacilli as therapeutics for dermal applications may be more rapid than would be the case for numerically dominant members of the of skin microbiota.

The skin forms a barrier between the entire body and the outside environment without which life as a terrestrial organism would be impossible. The epidermis of skin prevents both water loss from within the body and ingress of potential pathogens^9^. The importance of the skin barrier is exemplified in conditions where it is severely breached. For example, morbidity and mortality in victims of severe burns are associated with the dehydration and infection that occurs due to the poor skin barrier in these conditions^10^. However, as well as these extreme situations, breaches in the skin barrier can occur in daily life due to wounding and in surgery^11^. In healthy individuals, wounds normally heal in a timely fashion^12^. However, due to underlying conditions such as diabetes, some wounds heal slowly or not at all. In these cases, infection is a potential complication that can then further inhibit wound healing^13^,^14^. Chronic wounds are therefore a significant pathology. It follows then that treatments that can promote the wound healing process will be of considerable benefit to patients.

In the gut, certain species of probiotic bacteria have been demonstrated to increase barrier repair in *in vitro* and *vivo* models^15^,^16^. For example, healing of acid-induced gastric ulcers in the rat is reportedly accelerated in the presence of *L. rhamnosus* GG and *L. gasseri*^16^. Because of their purported effectiveness at promoting the healing of gastric wounds, attention has begun to focus on whether probiotic bacteria can impact upon the healing of cutaneous wounds. A recent study in rabbits showed that patches containing *L. fermentum* accelerated wound healing^17^. *L. plantarum* has been shown to improve healing of burns in mice and humans in a limited number of studies and also reportedly promotes healing of leg ulcers in humans^17^,^18^,^19^,^20^. However, in general, the mechanisms underlying these effects have not been fully explored.

We hypothesised that bacterial lysates would represent a safer alternative to the use of live bacteria in a wound situation because although probiotic lactobacilli have a GRAS (generally regarded as safe) status for food, the potential risks of live probiotic entering the bloodstream through breached skin has not been assessed. Furthermore, the use of lysates may be of more utility to potential wound care manufacturers than live bacteria because the logistical requirements of maintaining viability of bacteria within a formulation or wound dressing can be bypassed. In this study therefore, we have compared the ability of lysates made from four different species of lactobacilli to accelerate an important aspect of wound healing, re-epithelialization. To this end the scratch assay a well-established wound model^21^, was used to assess the effect of *Lactobacillus* lysates on re-epithelialization of human primary keratinocytes, the main cell type present within the epidermal layer of skin.

## Results

### Bacterial lysates accelerate scratch closure in a species dependent manner

**Figure 1:**
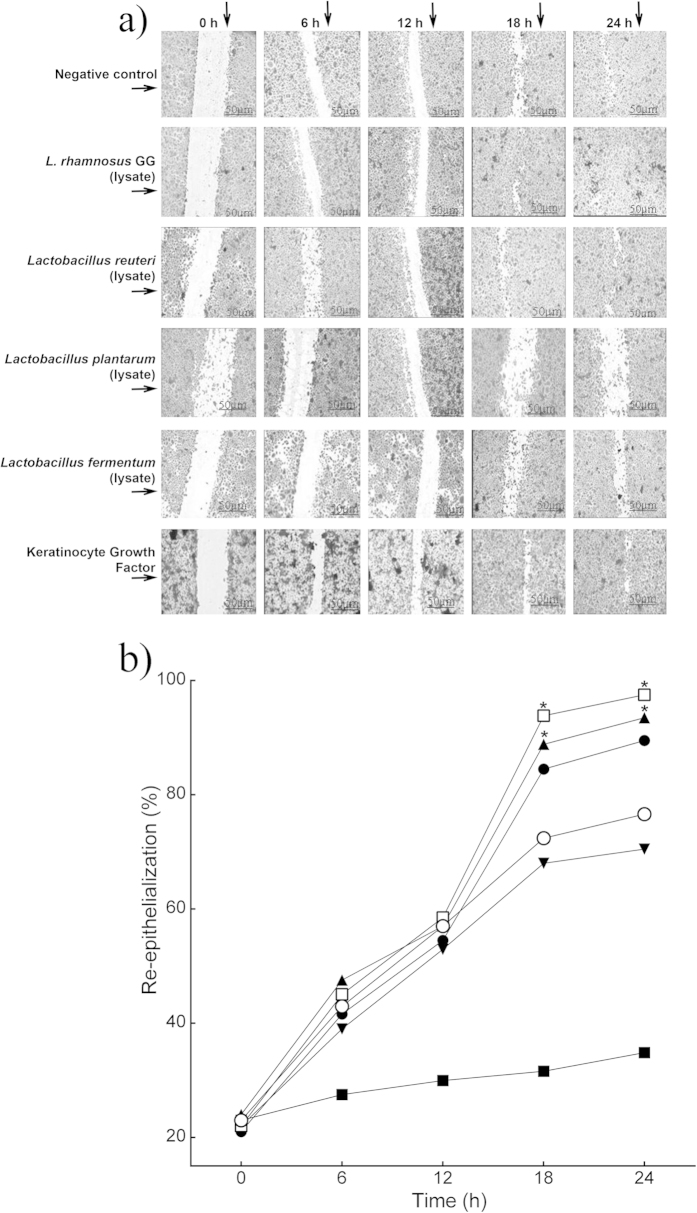
Specific probiotic lysates stimulate keratinocyte re-epithelialization *in vitro*. (**a**) Representative images of monolayer re-epithelialization in the presence/absence of different treatments. (**b**) The graph shows the percentage of scratch re-epithelialization in cultures treated with/without probiotic lysates at different time points. Results are expressed as the mean ± SEM. *P < 0.05. (○), control, untreated; (□), *L. rhamnosus* GG lysate; (●), *L. reuteri* lysate; (▼), *L. plantarum;* (■), *L. fermentum* lysate; (▲), keratinocyte Growth Factor.

Re-epithelialization in keratinocyte monolayers treated with *L. rhamnosus* GG lysate was significantly accelerated compared to that of untreated monolayers. At 18 h, 95% (*P* = 0.03, n = 3) of the scratch area was re-epithelialized compared with 75.2% in the control monolayer (Fig. 1a,b). Similar results were found when scratched cells were subjected to treatment with *L. reuteri* lysate, where 90.4% (*P* = 0.05, n = 3) of the scratch area was closed at 18 h (Fig. 1a,b). In contrast, *L. plantarum* lysate did not stimulate monolayer re-epithelialization (70.5%, n = 3). *L. fermentum* lysates caused a significant reduction in monolayer re-epithelialization rate compared with the control (40%, *P* = 0.01, n = 3). In keeping with the literature, the positive control, Keratinocyte growth factor (KGF) stimulated re-epithelialization of scratches although it was no more efficacious than the *L. rhamnosus* GG lysate (Fig. 1).

### Bacterial lysates increase keratinocyte migration and proliferation

**Figure 2:**
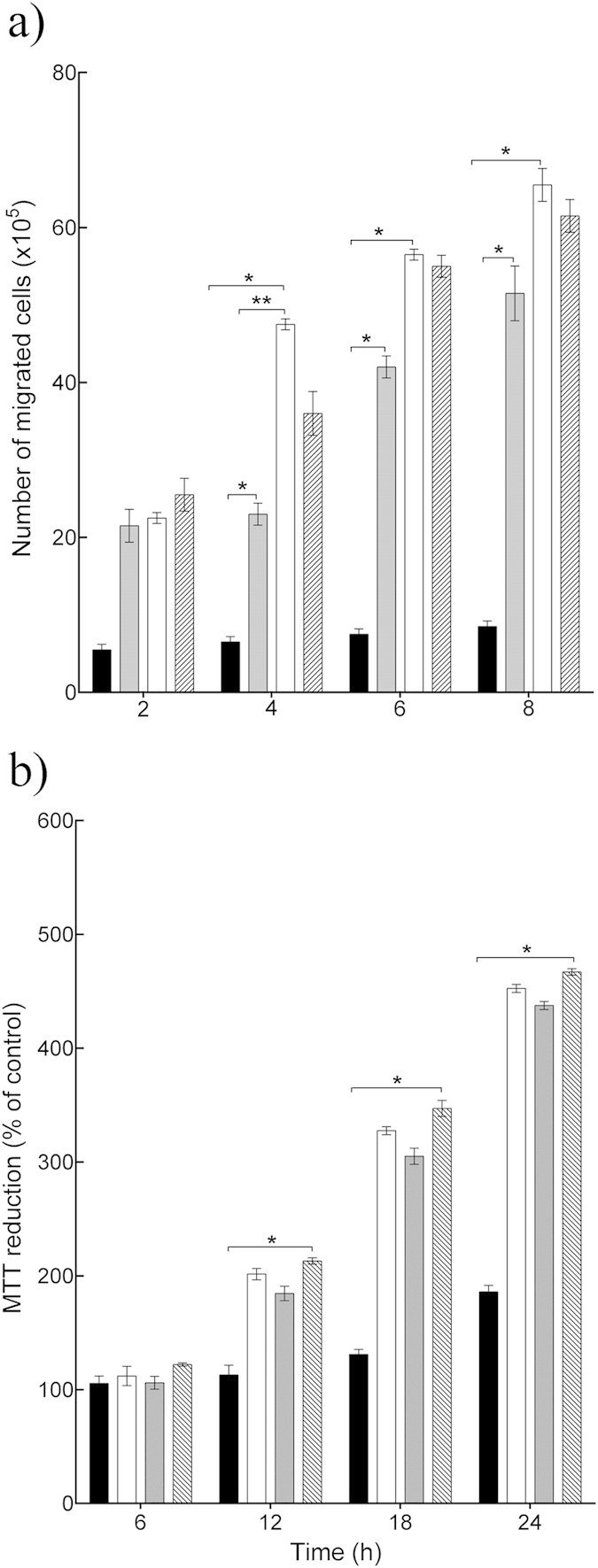
*L. rhamnosus* GG lysate and *L. reuteri* lysate increased keratinocyte migration and proliferation. (**a**) *L. rhamnosus* GG lysate and *L. reuteri* lysate significantly increased the number of migrated cells (*P* = 0.001, *P* = 0.004, respectively n = 3) after 4 h incubation. However, *L. rhamnosus* GG lysate was more efficacious than *L. reuteri* lysate (**P = 0.002, n = 3) and had similar effect to Keratinocyte Growth Factor. (**b**) *L. rhamnosus* GG lysate and *L. reuteri* lysate significantly increased cell proliferation after12h incubation. Results are expressed as the mean ± SEM, *P < 0.05. (■), control; (□) *L. rhamnosus* GG lysate; (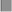), *L. reuteri* lysate; (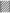), keratinocyte Growth Factor.

Since only *L. rhamnosus* GG and *L. reuteri* were efficacious in the scratch assay, these two lysates were analyzed further. Re-epithelialization could be due to keratinocyte migration and/or proliferation. Hence, the effect of *L. rhamnosus* GG and *L. reuteri* lysates on these keratinocyte activities was tested alongside that of the positive control KGF. In cultures treated with *L. rhamnosus* GG lysate and *L. reuteri* lysate there were about 48 × 10^5^ and 24 × 10^5^ migrated cells respectively after 4 h (*P* = 0.001, *P* = 0.004, respectively, n = 3), compared with 7 × 10^5^ cells in control cultures (Fig. 2a). Importantly, there was a significant difference between two lysates (*P* = 0.002, n = 3), with *L. rhamnosus* GG lysate being the most efficacious (Fig. 2a) in stimulating cell migration. KGF stimulated 34 × 10^5^ cells to migrate. However, there was no significant difference between the effects of KGF and *L. rhamnosus* GG lysate on keratinocyte migration. Incubation of keratinocytes with 100 μl of *L. rhamnosus* GG lysate or *L. reuteri* lysate resulted in significant proliferation as double the number of cells was found in these cultures relative to control cultures (*P* = 0.02, *P* = 0.03, respectively, n = 3) at 12 h post-inoculation (Fig. 2b). KGF caused a two-fold increase in the number of cells compared with the untreated control (Fig. 2b). Interestingly, both lysates and KGF afforded equal stimulation of proliferation.

### *L. rhamnosus* GG induces cell migration as the dominant mechanism

**Figure 3:**
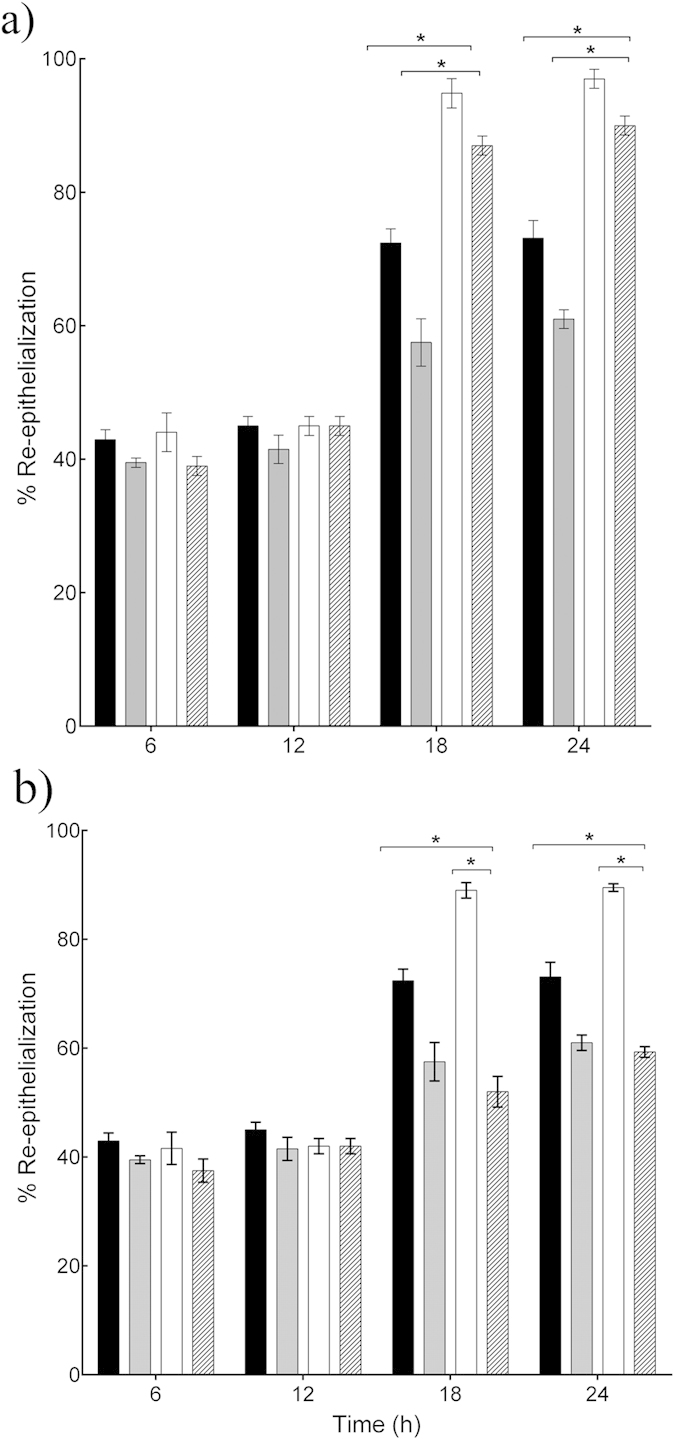
*L. rhamnosus* GG and *L. reuteri* enhance re-epithelialisation by stimulating migration and proliferation of keratinocytes. (**a**) *L. rhamnosus* GG lysate accelerated re-epithelialisation of scratches even in the presence of Mitomycin C (*P = 0.03, n = 3) after 18h incubation. (**b**) However, in cells treated with *L. reuteri* lysate and Mitomycin C, the percentage of re-epithelialisation was reduced significantly to 52% ± 2.32 at 18 h, compared to 90% ± 1.13 in cells treated with *L. reuteri* lysate alone (*P* = 0.01, n = 3) at 18 h. Results are expressed as the mean ± SEM, *P < 0.05. (■), control; (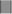), control and Mitomycin C; (□), *L. reuteri* lysate; (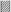), *L. reuteri* lysate and Mitomycin C.

Since the *L. reuteri* and *L. rhamnosus* GG lysates both stimulated migration and proliferation of keratinocytes, we next asked which of these mechanisms was most dominant in the acceleration of re-epithelialization induced by the two lysates. The rate of re-epithelialization of scratched keratinocyte monolayers was measured in the presence of the well-characterised inhibitor of cell proliferation, Mitomycin C. The data in Fig. 3a demonstrate that in monolayers treated with *L. rhamnosus* GG lysate and Mitomycin C, 94.5% of the scratch area re-epithelialized after 18h incubation (*P* = 0.03, n = 3) which was not significantly different to the re-epithelialization of monolayers treated with *L. rhamnosus* GG alone. However, in the presence of Mitomycin C the *L. reuteri* lysate*-* treated monolayer was only 52% re-epithelialized compared to 90% in cells treated with *L. reuteri* lysate alone (*P* = 0.01, n = 3, Fig. 3b).

### Genome-wide Affymetrix microarray study of the effects of the *L. rhamnosus* GG lysate on keratinocyte gene expression

Our data suggest that the *L. rhamnosus* GG lysate increases re-epithelialization by enhancing keratinocyte migration. Therefore, the pathways underlying this effect were explored using microarray analysis of scratched keratinocytes treated vs. untreated with *L. rhamnosus* GG lysate. The genes identified in the microarray were grouped using IPA according to cellular function. Table 1 considers functions known to be relevant to the wound-healing process^10^,^22^,^23^. The groups of genes involved in ‘cell movement’, ‘migration’ and ‘proliferation’ had the highest *P* value suggesting modulation of these genes by the bacterial lysate. The group of genes that was most significantly altered in expression was ‘cell movement’. However, all the genes contained within this group were also common to the group called ‘cell migration’. More than 1760 genes involved in migration, were altered in response to the *L. rhamnosus* GG lysate. The top 10 genes from the common group between ‘migration’ and ‘cell movement’ are presented in Table 2.

**Figure 4:**
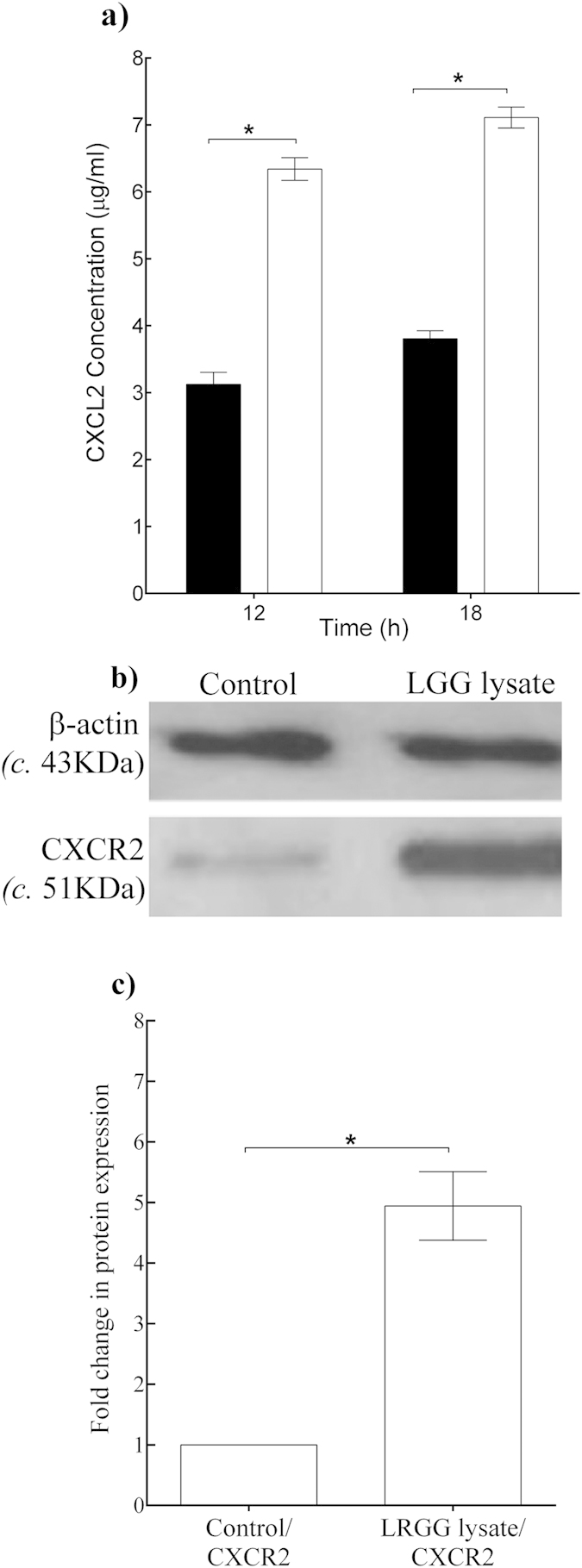
*L. rhamnosus* GG lysate increased the protein levels of CXCL2/CXCR2 in scratched keratinocyte cultures. (**a**) Untreated control cultures produced 3.10 μg/ml ± 2.32 of CXCL2 whereas treated cultures produced significantly higher concentrations of CXCL2, compared with untreated controls (*P* = 0.002, n = 3) after 12 h incubation. (**b**) Representative images of immuno-blots for CXCR2. (**c**) Densitometry analysis of the immunoblots was performed to quantify the change in protein levels for treated cell cultures vs. untreated controls (control). Changes were normalized against β-actin. *L. rhamnosus* GG lysate increased CXCR2 protein level compared to untreated control (*P* = 0.0009, n = 3). Data are representative of three individual experiments and are expressed as the mean ± SEM, *P < 0.05. For (**a**) (■), control; (□) *L. rhamnosus* GG lysate.

Table 2 shows that the expression of two genes, CXCL2 and its receptor CXCR2 were highly up-regulated by the *L. rhamnosus* GG lysate. The microarray was validated by performing quantitative PCR analysis of CXCL2 and CXCR2 mRNA levels in scratched keratinocytes treated with *L. rhamnosus* GG lysate vs untreated controls (data not shown). Since the *L. rhamnosus* GG lysate up-regulated the gene expression of CXCL2, and its corresponding receptor, we next investigated whether the proteins corresponding to these genes were also increased by the lysate. The secretion of CXCL2 by scratched keratinocytes in response to *L. rhamnosus* GG lysate after 12 h and 18 h incubation was measured using ELISA. These showed that increased CXCL2 secretion was stimulated by *L. rhamnosus* GG lysate-treated keratinocytes, with 6.30 ± 0.4 μg/ml, compared with 3.10 ± 0.7 μg/ml in untreated cells after 12h incubation (*P* = 0.002, n = 3, Fig. 4a). Immunoblot analysis was performed to quantify the changes in the levels of CXCR2 protein receptors in cells 12 h post-treatment with *L. rhamnosus* GG lysate. Analysis of extracts demonstrated that *L. rhamnosus* GG lysate caused an up-regulation of CXCR2 protein levels amounting to four times greater than in untreated cells (*P* = 0.0009, n = 3, Fig. 4b,c).

## Discussion

Non-healing wounds such as diabetic foot ulcers are a significant cause of morbidity and can lead to mortality^24^. Over-use of antibiotics has given rise to many antibiotic-resistant infections that are frequently associated with poor outcomes in wound healing, especially following surgery^25^. Currently, there is an unmet, clinical need for new therapies in the treatment of wounds. Since probiotics have been demonstrated to promote wound healing in the gut^15^, the aim of this study was to examine the effects of enteric probiotic lysates on keratinocyte migration and proliferation that are important aspects of the wound healing response.

Lysates of *L. rhamnous* GG and *L. reuteri* promoted the re-epithelialization of keratinocyte monolayers. In contrast, lysates from the other lactobacilli tested either had no effect on re-epithelialization, (*L. plantarum*) or inhibited re-epithelialization (*L. fermentum*). This latter result is probably related to our previous observation that *L. fermentum* reduces keratinocyte viability^6^,^8^. A previous study examined the effects of cell-free supernatants of *L. plantarum* on the wound healing process especially, the proliferation and migration of human keratinocytes *in vitro*^26^. The study demonstrated that the *L. plantarum* cell free supernatant promoted re-epithelialization in keratinocyte cultures. It was hypothesized that *L. plantarum* produces a specific substance, Plantaricin A that stimulates keratinocyte migration and proliferation in a scratch assay^26^. The authors investigated the mechanism underlying improved migration and proliferation and showed increased expression of transforming growth factor (TGF-β1), vascular endothelial growth factor (VEGF) and fibroblast growth factor (FGF-7). In the present study, the effect of spent culture fluid of *L. plantarum* on the stimulation of keratinocyte re-epithelialization was not measured. Therefore, it is not possible to exclude the likelihood that the spent culture fluid from *L. plantarum* may have been efficacious in our assay. What is clear is that lysates of both *L. reuteri* and *L. rhamnosus* GG are more efficacious than the *L. plantarum* lysate in promoting keratinocyte proliferation/migration.

The enhanced re-epithelialization induced by *L. rhamnosus* GG and *L. reuteri* was probably due to increased keratinocyte migration and proliferation although the two lysate were not equally effective in promoting cell migration. *L. rhamnosus* GG was much more effective at stimulating migration than *L. reuteri*. Indeed, migration is probably the dominant mechanism promoted by *L. rhamnosus* GG because in the presence of Mitomycin C, scratches were still able to re-epithelialize at a significantly faster rate than untreated scratches. Conversely, the dominant mechanism for the *L. reuteri* lysate was probably stimulation of proliferation, because Mitomycin C completely negated the stimulatory effects of the *L. reuteri* lysate. This suggests that lysates from different *lactobacilli* have species-specific effects on keratinocyte functions and is in agreement with previous observations^6^,^7^,^8^.

The mechanism underlying the increase in re-epithelialization induced by *L. rhamnosus* GG potentially involves increased expression of the chemokine, CXCL2 and its receptor, CXCR2. The expression of these genes was greatly enhanced at both the mRNA and protein levels as demonstrated by microarray, q-PCR, ELISA and immunoblotting data. CXCL2 is known to have several roles in the wound-healing process such as the stimulation of keratinocyte migration, proliferation and adhesion^27^,^28^. Indeed, it has been suggested that CXCL2 may be a chemoattractant for human keratinocytes^27^,^28^. Moreover, a number of *vitro* studies have shown that CXCL2 stimulates the production of additional chemokines, such as CXCL12 and CXCL10 also act as chemoattractants for keratinocytes in the wound-healing process^26^,^29^,^30^. The CXCL2 receptor, CXCR2 is also known to be important to the wound healing process. Mice lacking CXCR2 have delayed wound healing and this was related to a delay in cell recruitment to the wound site and a delay in re-epithelialization^26^,^29^,^30^. Based on these findings and the present data, we hypothesize that *L. rhamnosus* GG lysate increases re-epithelialization potentially via the interaction of CXCR2 and CXCL2 that accelerates keratinocyte migration, although other factors could also be involved.

A study conducted in 2011 by Polk^31^ reported that material derived from *L. rhamnosus* GG regulated intestinal epithelial cell survival and growth in injury via stimulating epidermal growth factor (EGF) and its receptor^31^,^32^. The microarray data in the present study did not highlight a role for EGF nor its receptor alpha chain EGFR-α since the gene levels of neither of these were significantly altered in cells treated with *L. rhamnosus* GG lysate. However, some of the main genes in the EGF signaling cascade, such as EGFR-β, MEK1, STAT3 and ADAM17, were significantly up regulated in response to *L. rhamnosus* GG lysate. CXCL2 and CXCR2 were both significantly upregulated and higher than the expression of genes associated with the EGF pathway. Polk’s study^32^ also identified specific proteins from *L. rhamnosus* GG spent medium fluid that promoted cell growth in cultured human and mouse colon epithelial cells. In the current study, the spent culture fluid was tested but found not to be as efficacious as the lysate (Figure S1). It is possible that this is due to a concentration effect i.e. the effective concentration is not sufficiently high in the spent culture fluid for an effect on re-epithelialization to be observed. Conversely, the *L. rhamnosus* GG lysate may be working via different receptors and ligands in keratinocytes than has been reported for enterocytes^29^,^30^,^31^.

In conclusion, *L. rhamnosus* GG lysate is a potential new wound healing treatment. We have demonstrated previously^6^ that this lysate also has protective effects against *S. aureus* toxicity towards keratinocytes. Thus, it is possible that *L. rhamnosus* GG lysate may have utility in the treatment of infected, non-healing wounds.

## Materials and Methods

### Bacterial cell culture

*Lactobacillus rhamnosus* GG (ATCC 53103) *Lactobacillus reuteri* (ATCC55730), *Lactobacillus plantarum* (UCC118) (ATCC10241), and *Lactobacillus fermentum* (ATCC14932) were purchased LGC Ltd, Ireland, UK. These were grown routinely in Wilkins-Chalgren Broth or Agar (Oxoid, Basingstoke, UK) at 37 °C incubated in an anaerobic cabinet (atmosphere, 10:10:80, H_2_-CO_2_-N_2_). Lysates of all these species were prepared as previously described^6^.

### Primary keratinocyte culture and scratch assay

Primary human epidermal keratinocytes were cultured as described in^7^. For scratch assays, keratinocytes were plated on 24 well tissue culture plates and allowed to reach confluence. At this point a scratch was introduced into the monolayer using a sterile p200 pipette tip. The cells were then washed in phosphate buffered saline (0.01M PBS, pH = 7.4) to remove the debris and the medium was replaced with either fresh medium (control samples) or medium containing 100 μl bacterial lysate (0.93 mg/μl). In some experiments, keratinocyte growth factor (KGF, 0.5 mg/ml) was included as a positive control.

In other experiments, 0.5 mg/ml Mitomycin C from *Streptomyces caespitosus* was added before the addition of the lysate to inhibit cell proliferation. Scratches were then monitored and documented at 0,6,12,18 and 24 h post scratch by staining cells with crystal violet solution. Images were captured under the Keyence microscope (x5 magnification, Keyence, Osaka, Japan) and analysed using IMAGE J-64 software (http://imagej.nih.gov). This enabled determination of the percentage of scratch area (gap) with respect to the percentage of starting scratch area at time zero. For each image, the area between one side of the scratch and the other was measured. Quantification of scratch closure was performed by comparing the area of the scratch at set time points according to the equation:


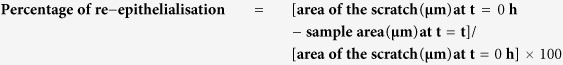


where t **=** t is a specific time point post scratching. The experiment was performed three times with triplicate samples within each individual experiment by using different keratinocytes donors.

### Keratinocyte migration assays

A migration assay was performed by plating *c*. 2.5 × 10^5^ cells per well in the upper wells of a 24-Transwell^TM^ chamber (Invitrogen, Life Technologies Ltd, Paisley) and then the 100 μl bacterial lysate under test, or the positive control, keratinocyte growth factor (KGF, 0.5 mg/ml) was placed in the lower chamber. The chambers were separated by an 8 μm pore-size permeable membrane that allowed the keratinocytes to move down toward chemo-attracting lysates. At 2, 4, 6 and 8h post inoculation, the membrane was stained with crystal violet to visualise the cells. Non-migrating cells in the upper surface were removed, whereas cells adhering to the lower surface of the membrane were considered as having migrated. The number of migrated cells was determined by counting the cells in three high powered fields (images were captured at 40 × magnification; bars = 100 μm) and this number was subtracted from the initial number of cells seeded i.e. 2.5 × 10^5^. The experiment was performed three times with triplicate samples within each individual experiment.

### Keratinocyte proliferation assay

The proliferation assay (CellTiter 96® AQueous) was performed according to the manufacturer’s instructions (Promega, Madison, USA). Cells were seeded at an initial density of 2.5 × 10^5^ cells per well in 96-well plates. After 24 h incubation, cells were exposed to 100 μl of probiotic lysates or to the positive control, KGF at 0.5 mg/ml. Plates were then incubated at 37 °C, for 6, 12, 18 and 24 h. Once each time point was reached, the medium was aspirated and replaced with 100 μl per well of the CellTiter 96® Aqueous reagent. After 3 h of incubation, the absorbance of the solutions was measured at 490 nm in a Titertek micro-plate reader (Flow Laboratories Ltd. Leyland, UK). The absorbance of cells in the lysate-treated wells was compared with that in their untreated counterparts. The absorbance of 2.5 × 10^5^ cells (original seeding number) was taken as the control absorbance and that of treated cells was normalized to this control. The experiment was conducted on three separate occasions with triplicate samples within each individual experiment.

### Micro-array analysis of scratch assays

Confluent keratinocytes were scratched and exposed to bacterial lysate for 12 h at 37 °C in 5% CO_2_. Cells were washed twice in phosphate buffered saline (0.01M PBS, pH = 7.4) and the total RNA was extracted from treated and untreated control cells using 1.0 ml Trizol (Invitrogen, Life Technologies Ltd, Paisley, UK) per 10 cm^2^ of the culture dish. RNA was purified using an Ambion PureLink® RNA Mini Kit according to the manufacturer’s instructions (Invitrogen, Life Technologies Ltd, Paisley). The samples gathered from three individual extractions were analysed on an Agilent 2100 Bioanalyzer Instruments (Agilent Technologies, CA, United States) using the Human Genome U133 plus 2.0 Affymetrix GeneChips according to the manufacturer’s instructions. Bioinformatics analysis was performed with dChip (V2005) (www.dchip.org). The model-based analysis of oligonucleotide arrays and gene expression analysis were carried out using Bioconductor as described in^33^. Filtering for probe sets with a *P* ≤ 0.05 created a list of differentially expressed genes. *P* value refers to the alteration in gene expression in treated vs untreated cells. Data was analysed using the Ingenuity Knowledge Base program (IPA, http://www.ingenuity.com/products/ipa) with the aim of identifying the genes related to the wound-healing process. The microarray data was submitted to the array express repository, ID=E-MTAB-3485.

### Analysis of chemokine production

A scratch assay was performed and the cells treated with or without lysate as described above. At 12 h or 18 h after scratching, the spent cell culture fluid yielded from the plates was removed. The CXCL2 level was determined by ELISA (Invitrogen, Life Technologies Ltd, Paisley, UK), in adherence to the manufacturer’s instructions. The optical density of wells was determined using a Labtek LT400 microplate reader (Labtech International Ltd, UK) set to read absorbance at 450 nm. The experiment was carried out on three separate occasions with triplicate samples within each individual experiment.

### SDS-PAGE and Immunoblotting

This was performed as described previously^8^ using primary antibodies CXCR2/IL-8 RB (2.5 μg in1 ml of PBS) overnight at 4 °C. Membranes were washed for 3 × 5 minutes with TBS/Tween containing 5% skimmed milk solution and then incubated with secondary antibody (HRP-conjugated goat anti-mouse IgG) at 1:5000 dilutions for 1h at room temperature. Then the immunoblotting assay was performed as described in^8^.

### Statistical analyses

All experiments were performed three times with triplicate samples within each individual experiment. For experiments comparing two or more treatments, a two-way ANOVA with post hoc Tukey test were utilised to analyse the main effects of, and interactions between, multiple factors. Results were considered significant if *P* ≤ 0.05, and all analysis were performed using the SPSS (IBM SPSS Statistics version 16.0) program.

## Additional Information

**How to cite this article**: Mohammedsaeed, W. *et al.* *Lactobacillus rhamnosus* GG Lysate Increases Re-Epithelialization of Keratinocyte Scratch Assays by Promoting Migration. *Sci. Rep.* **5**, 16147; doi: 10.1038/srep16147 (2015).

## Supplementary Material

Supplementary figure s1

## Figures and Tables

**Table 1:** Bioinformatics analysis of the effects of *L. rhamnosus* GG lysate on keratinocyte activities.

Function	Overall p-value	Number of molecules
Cell movement	9.35E-06	90
Migration	8.22E-06	391
Proliferation	5.31E-06	1372
Mitosis	1.91E-06	55
Cell cycle progression	6.77E-05	56
Antimicrobial response	4.10E-05	11
Transdifferentiation	4.55E-04	3
Cellular Growth	2.81E-04	26
Transmigration of cells	2.28E-04	15
Apoptosis	7.69E-03	163
DNA Replication, Recombination, and Repair	7.67E-03	18
Differentiation	7.01E-03	65
Cell transformation	5.52E-03	14
Synthesis of protein	4.55E-03	18
Adhesion of cells	4.29E-03	25
Bacterial Infection response	3.99E-03	3
Generation of reactive oxygen	1.80E-03	8
Activation of cells	4.29E-02	6
Chemotaxis of cells	4.16E-02	16
Generation of superoxide	4.45E-02	3
Cytostasis	4.33E-02	10
Trans-membrane potential	4.29E-02	6
Angiogenesis	3.79E-02	26
Activation of antigen presenting cells	3.53E-02	11
Aggregation of cells	3.12E-02	4
Cell signalling	2.52E-02	170
Inflammatory Response	2.45E-02	14
Mobilization of Ca^2^+	1.61E-02	16
Attachment of cells/Tight junction formation	1.29E-02	25

A summary of the cellular biological processes related to the wound healing process, corrected *P*-values and the number of associated molecules affected by *L. rhamnosus* GG lysate. The change in gene expression in cells treated with lysate were considered significant when the overall *P*-value was <0.05, compared with the control. The data represent three individual experiments.

**Table 2:** *L. rhamnosus* GG modulates the expression of genes involved in cell migration.

Gene	Fold change	p-value
CXCL2	19.899	9.12E-10
CXCR2	10.18	1.48E-04
CCL20	4.505	0.0012
EDN2	5.698	0.001
TNFRSF18	5.80	0.001
IGFBP3	4.550	0.002
TNF	10.05	0.004
CXCL10	4.427	0.006
Cortactin	3.00	0.01
MAP3K13	3.193	0.001

The top 10 genes modulated by the lysate that are involved specifically with cell migration, their corresponding *P* values and fold changes. The change in gene expression in cells treated with lysate were considered significant when *P*-value was <0.05 and the fold change was greater than two compared with control (n = 3).
