# Author Correction: *Lactobacillus rhamnosus* GG lysate increases re-epithelialization of keratinocyte scratch assays by promoting migration

**DOI:** 10.1038/s41598-024-52533-2

**Published:** 2024-01-25

**Authors:** Walaa Mohammedsaeed, Sheena Cruickshank, Andrew J. McBain, Catherine A. O’Neill

**Affiliations:** 1 Institute of Inflammation and Repair, The University of Manchester, Manchester, UK; 2 Faculty of Life Sciences, The University of Manchester, Manchester, UK; 3 Manchester Pharmacy School, The University of Manchester, Manchester, UK

Correction to: *Scientific Reports* 10.1038/srep16147, published online 05 November 2015

This Article contains an error in Figure 1a.

As a result of an error during this figure assembly, *L. reuteri* at 12h is a duplication of *L. plantarum* at 12h. Additionally, the panel labelled ‘*L plantarum* should be *labelled L. fermentum* and vice versa.

The correct version of Figure 1a is shown below.

**Figure 1:**
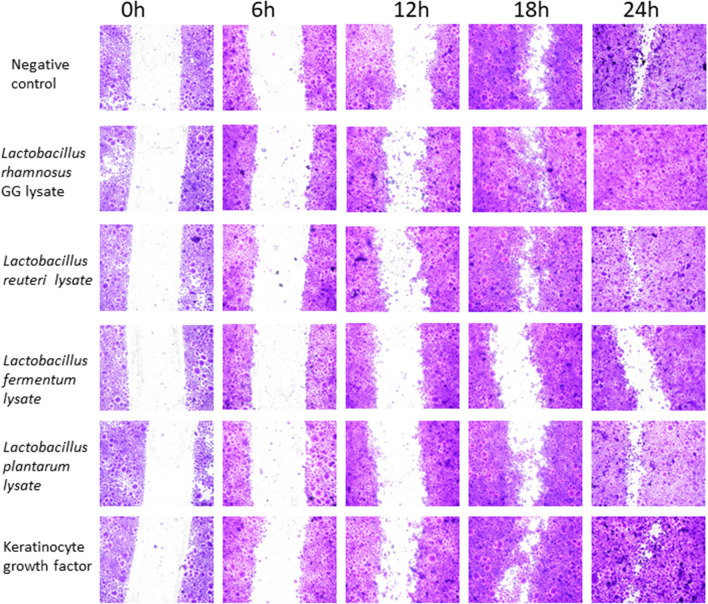
Specific probiotic lysates stimulate keratinocyte re-epithelialisation in vitro. Representative images of monolayer re-epithelialisation in the presence/absence of different treatments.

