# Supplementary material for: Lactobacillus rhamnosus GG Lysate Increases Re-Epithelialization of Keratinocyte Scratch Assays by Promoting Migration
Source: Sci Rep. 2015 Nov 5;5:16147. doi: 10.1038/srep16147 (PMC4633615; doi:10.1038/srep16147)
Supplement: Supplementary figure s1 [file srep16147-s1.doc]

***Lactobacillus rhamnosus* GG Lysate Increases Re-Epithelialization of Keratinocyte Scratch**

**Assays by Promoting Migration**

Walaa Mohammedsaeed^1^, Sheena Cruickshank^2^,

Andrew J. McBain^3^ and Catherine A. O’Neill^1^

Institute of Inflammation and Repair^1^, Faculty of Life Sciences^2^ and Manchester Pharmacy School^3^, The University of Manchester, Manchester U.K.


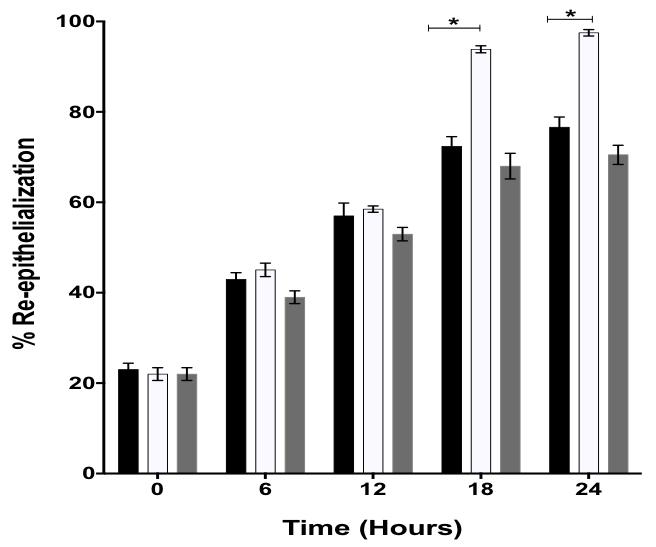


**Figure S1: *L.rhamnosus* GG lysate but not its spent culture fluid stimulates keratinocyte re-epithelialization in *vitro*.** The graph shows the percentage of scratch re-epithelialization in cultures treated with/without probiotic lysates at different time points. *L. rhamnosus* GG lysate (white bars) significantly accelerated re-epithelialization compared to untreated monolayers (black bars). At 18h, 95% (*P*=0.03, n=3) of the scratch area was re-epithelialized compared with 75.2% in the control monolayer. *L. rhamnosus* GG spent culture fluid (gray bars) showed no stimulation of monolayer re-epithelialization (77.5%, n=3). Results are expressed as the mean ± SEM, **P*<0.05.
